# Evaluation of the temperature and humidity index to support the implementation of a rearing system for ruminants in the Western Amazon

**DOI:** 10.3389/fvets.2023.1198678

**Published:** 2023-07-14

**Authors:** Welligton Conceição da Silva, Oscar Vitor Nina Printes, Dagmar Oliveira Lima, Éder Bruno Rebelo da Silva, Maria Roseane Pereira dos Santos, Raimundo Nonato Colares Camargo Júnior, Antônio Vinicius Corrêa Barbosa, Jamile Andréa Rodrigues da Silva, André Guimarães Maciel e Silva, Lilian Kátia Ximenes Silva, Cláudio Vieira de Araújo, Elton Nunes Britto, José de Brito Lourenço-Júnior

**Affiliations:** ^1^Postgraduate Program in Animal Science (PPGCAN), Institute of Veterinary Medicine, Federal University of Para (UFPA), Federal Rural University of the Amazon (UFRA), Brazilian Agricultural Research Corporation (EMBRAPA), Castanhal, Pará, Brazil; ^2^Department of Veterinary Medicine, University Center of the Amazon (UNAMA), Santarém, Pará, Brazil; ^3^Institute of Engineering and Geosciences, Federal University of Western Pará (UFOPA), Santarém, Pará, Brazil; ^4^Cyberspace Institute, Federal Rural University of the Amazon (UFRA), Belem, Pará, Brazil; ^5^Institute of Animal Health and Production, Federal Rural University of the Amazon (UFRA), Belem, Pará, Brazil; ^6^Institute of Veterinary Medicine, Federal University of Para (UFPA), Castanhal, Pará, Brazil; ^7^Department of Veterinary Medicine, Federal University of Mato Grosso, Sinop, Mato Grosso, Brazil; ^8^Department of Veterinary Medicine, Federal Institute of Pará (IFPA), Santarém, Pará, Brazil

**Keywords:** thermal stress, thermal comfort, climate, cattle, buffalo

## Abstract

The good productive and reproductive performance of the animals depends on multiple factors, including favorable climatic conditions, which are responsible for causing changes in the physiological and behavioral responses. Thus, the objective of this study was to evaluate the temperature and humidity index (THI) to support the implementation of a rearing system in ruminants in the Western Amazon, Brazil. Monthly temperature and relative humidity data were obtained from the Database for Teaching and Research (BDMEP) for the capitals Manaus (Amazonas), Boa Vista (Roraima), and Rio Branco (Acre), considering a historical series of 27 years (1993 to 2020), referring from January to December. In the capital Boa Vista, the months of January, February, May, June, and July showed an indication of mild stress and the months of March, April, August, September, October, November, and December had moderate stress. In Rio Branco, all months of the year presented the average THI in mild stress with variations of lower THI (73) and higher THI (77). In the capital Manaus, the months from January to July signaled mild stress, but from August to November, there was moderate stress, and in December, there was mild stress. It is possible to observe significant climatic variations during the months as well as the years of study, with the animals under thermal stress with THI > 72 or in a warning signal, with a gradual increase in temperature and humidity indices over the last 10 years. The importance of the breeding system and the consideration of environmental factors, such as the THI, are fundamental for the wellbeing and performance of cattle raised in the field. Our results support the adoption of heat stress mitigation strategies for ruminants in Western Amazon.

## 1. Introduction

Among the important conditions for raising production animals, regardless of the system applied to the herd, the climatic factor is essential to provide a comfortable environment. Considering this factor has positive effects on the metabolism and homeostasis of the animals, improving their degree of Animal Welfare (AW), in consequence, can maximize the productive performance of the herd, which in a comfortable environment can express their genetic qualities. Therefore, climate analyses are supervised as one of the planning actions for cattle ranchers before starting animal breeding (1–3).

In this context, climatic conditions can cause physiological and behavioral responses. For example, when temperature and humidity indices are high (THI above 72), they trigger thermal stress that causes changes in the biological functions of these animals (4–6), such as alterations in food intake, changes in enzymatic reactions, hormonal secretions, and immune status, which can impact productivity indices (7–10).

Heat stress refers to a condition in which an organism's body is exposed to excessive heat that exceeds its ability to dissipate heat and maintain normal regulatory functions (11). Despite the recognition of the harmful effects caused by heat stress on the animal organism that has occurred since the beginning of the century, studies about it persist until the present day with the aim of quantifying heat stress, as well as the most assertive way of measuring the physiological limits of animals during this process in order to optimize the animal response (12–15).

Heat stress in ruminants has consequences on their physiology and performance. These adverse conditions satisfied the ruminants' thermoregulation, which is the mechanism by which they maintained their body temperature within a normal range (15). In response to heat stress, ruminants may experience tachypnea, hyperthermia, and behavioral changes, such as seeking shade and reducing activity (15, 16).

The increase in environmental temperature and humidity interferes with the capacity of ruminants to dissipate the heat accumulated in their bodies. This leads to an increase in core body temperature and can cause regulatory dysfunctions such as changes in enzyme activity. Essential enzymatic reactions for food metabolism, such as the digestion and absorption of nutrients, can be compromised under conditions of heat stress, leading to a decrease in food intake and lower efficiency of feed conversion (16).

Furthermore, heat stress affects the hormones that regulate thermoregulation and the immune response in ruminants. Hormones, such as cortisol, produced by the adrenal gland in response to stress, can be influenced by temperature and affect the immune system of animals. This can lead to reduced immune response and increased susceptibility to disease and infection (17).

Studies on thermal comfort in the creation of ruminants by climatic elements are carried out through indices that evaluate different effects and have a relationship with the physiological and behavioral indicators of these animals (14, 18, 19).

The search for advances in research aimed at animal welfare, together with the mapping of the climatic conditions to which the animals are subjected, aims to direct more correct decision-making in relation to environmental management, and thereby minimize stress caused to animals by weather conditions (15, 20–22). In regions such as the Western Brazilian Amazon, where ruminant production systems are present, heat stress can pose a significant challenge. The increase in temperatures due to global warming may contribute to the more frequent occurrence of thermal stress conditions in these locations. In addition, the high relative humidity common in the region can further aggravate heat stress in ruminants.

Based on this information, the objective of this study was to evaluate the THI to support the implementation of a rearing system for ruminants (cattle, sheep, and goats) in the Western Amazon, Brazil.

## 2. Materials and methods

### 2.1. Study area

**Figure 1:**
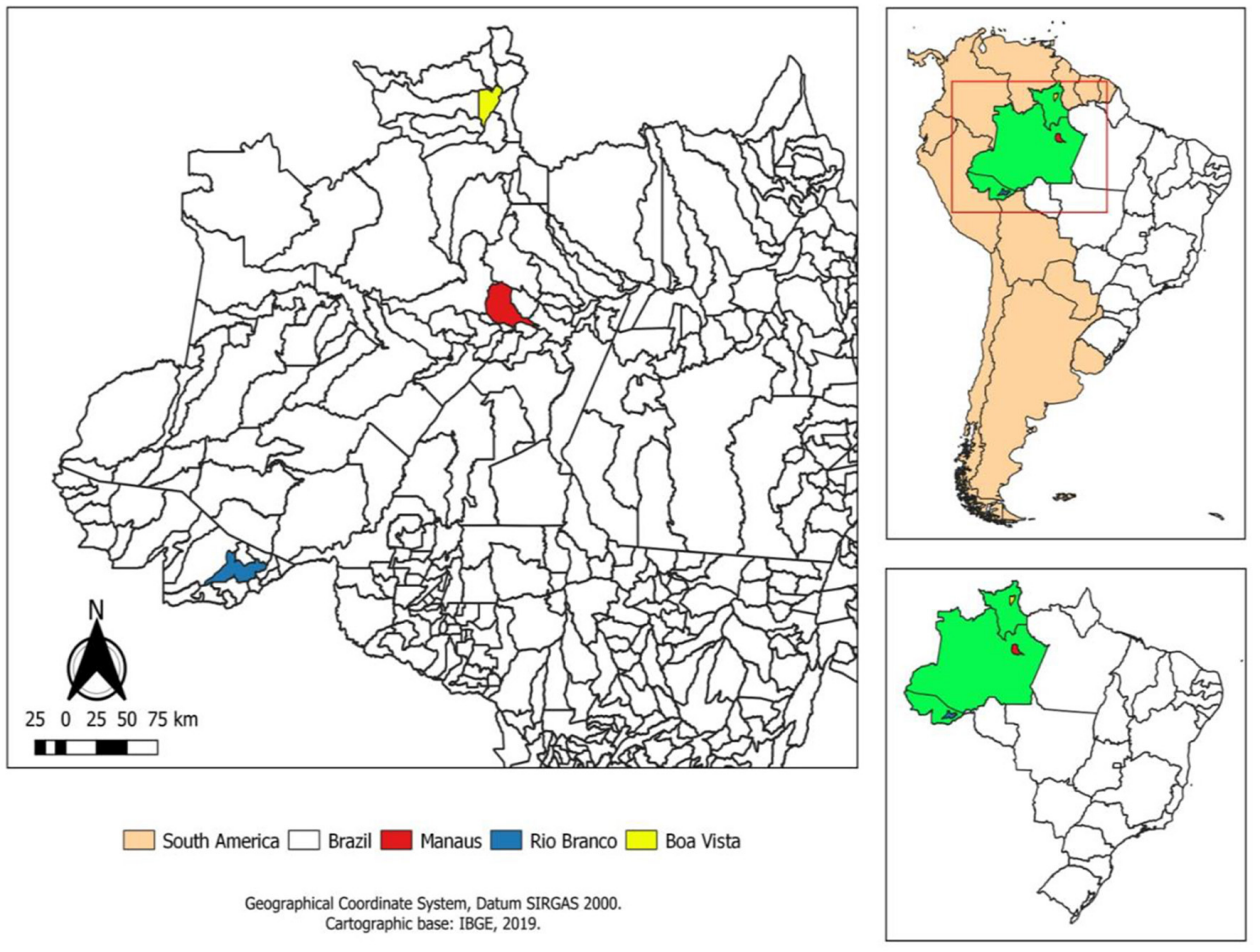
Location of the studied areas.

This study was carried out in three capitals in the northern region of Brazil, Manaus (Amazonas), Boa Vista (Roraima), and Rio Branco (Acre), located in the Western Amazon (Figure 1).

### 2.2. Climate information

The predominant climate in the region, according to the Köppen classification, is type Aw, tropical rainy, reaching annual averages of precipitation, relative humidity, and ambient temperature of 1,667 mm, 70%, and 27.4°C, respectively (23). The climate present in the research region located in the north of Brazil in the Western Amazon is classified as an equatorial climate according to the Köppen classification, presenting a hot and humid environment predominant throughout the Amazon region. In this region, there are two distinct seasons: a dry season, from June to August, and another rainy season, from October to April, with May and September being the transitional months. During the wet-test period, the relative humidity of the air is approximately 88%, and the daily oscillation varies from 55 to 98%. During the dry period, the average is 75%, and the daily variation is between 50 and 87%. Between August and October, the highest temperatures of the year occur, with maximum values between 33°C and throughout the year from 29 to 31°C (24).

### 2.3. Weather data

Data were collected through the digital platform of the National Institute of Meteorology (INMET), from conventional stations, registered with the Meteorological Organization (WMO), related to maximum temperature (Tmax), mean temperature (Tmed), minimum temperature (Tmin), maximum relative humidity (RHmax), mean relative humidity (RHmed), and minimum relative humidity (RHmin) for each month (January, February, March, April, May, June, July, August, September, October, November, and December) from 1993 to 2020, in three meteorological stations, specifically in Boa Vista (RR), Manaus (AM), and Rio Branco (AC), with registration numbers of 82024, 82331, and 82915, respectively.

### 2.4. Temperature and humidity index

The temperature and humidity index (THI) was calculated, taking into account temperature and relative humidity. The THI was obtained using equation 1 adapted from Thom (25).


$$\begin{array}{l} THI=\left(0,8^{*}T\right)+{\left(RH/100\right)}^{*}\left[\left(T-14,4\right)+46,4\right], \end{array}$$

where T is the dry bulb air temperature (°C) and RH is the relative air humidity (%).

To assess whether the ruminants (cattle, sheep, and goats) were under heat stress, the model described by Armstrong (26) was used, where < 72 indicates without stress; 72–78 indicates mild or mild stress; 79–88 indicates moderate stress; and 89–98 indicates severe stress.

### 2.5. Statistical analysis

The records of minimum, average, and maximum THI of each year were grouped into three periods as follows: 1993 to 1999, 2000 to 2009, and 2010 to 2020. Posterity was realized through statistical analysis using the Friedman test to compare between states, Roraima (RR), Acre (AC), and Amazonas (AM), in each period and to compare between periods (1, 2, and 3) in each state using the Kruskal–Wallis test. In all situations, the statistical significance level of the tests was a *p*-value of < 0.05. All analyses were performed using software R version 3.4.1 [R Core Team (27)].

## 3. Results

**Figure 2:**
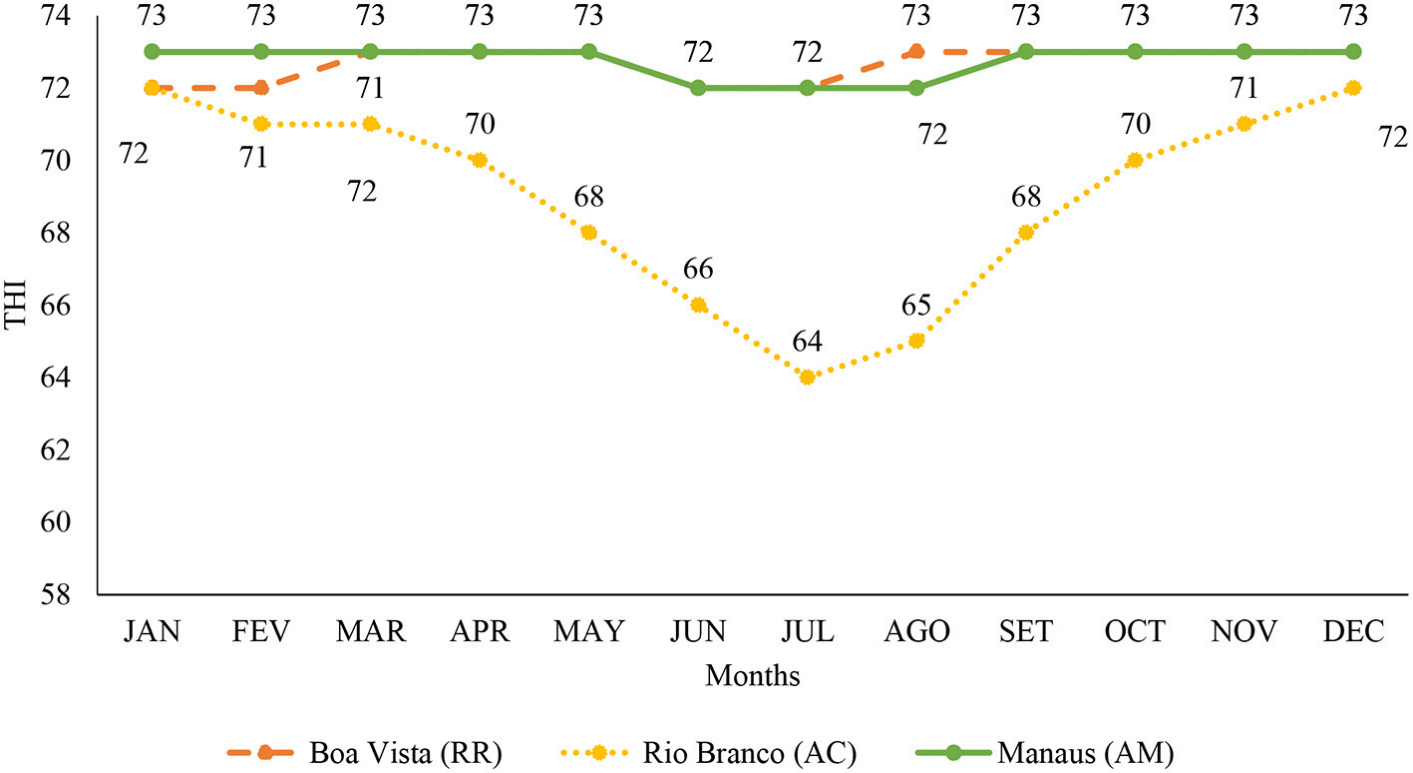
Minimum temperature and humidity index from January to December in the capitals of Roraima, Rio Branco, and Manaus, for the period from 1993 to 2020. <72 indicates without stress; 72–78 indicates mild or mild stress; 79–88 indicates moderate stress; 89–98 indicates severe stress (26). JAN, January; FEV, February; MAR, March; APR, April; MAY, May; JUN, June; JUL, July; AGO, August; SET, September; OCT, October; NOV, November; DEC, December.

In Figure 2, the variation of the minimum THI is observed, where the month of January presented an indication of mild thermal stress in the capital Rio Branco, from February to November presented thermal comfort, and December presented mild thermal stress, different from the capital Manaus, where it had mild stress during all months of the year. In the capital Boa Vista, mild stress was observed from January to December.

**Figure 3:**
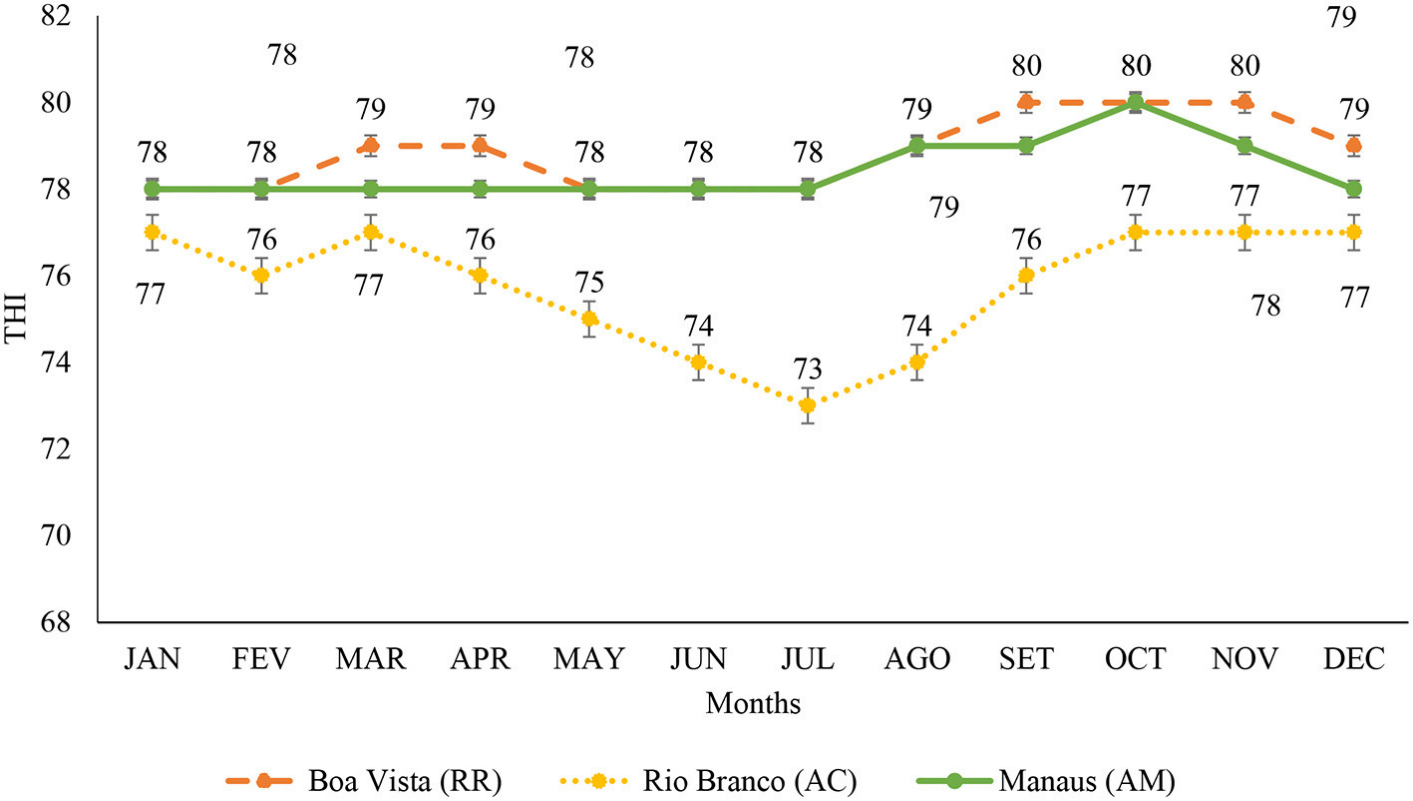
Average temperature and humidity index from January to December in the capitals of Roraima, Rio Branco, and Manaus, for the period from 1993 to 2020. <72 without stress; 72–78 indicates mild or mild stress; 79–88 indicates moderate stress; 89–98 indicates severe stress (26). JAN, January; FEB, February; MAR, March; APR, April; MAY, May; JUN, June; JUL, July; AGO, August; SET, September; OCT, October; NOV, November; DEC, December.

Figure 3 shows the average THI variation, with thermal stress being observed in all months of the year, classified as mild stress and moderate stress. In the capital Boa Vista, the months of January, February, May, June, and July showed an indication of mild stress and the months of March, April, August, September, October, November, and December had moderate stress. In Rio Branco, all months of the year presented the average THI in mild stress with variations of lower THI (73) and higher THI (77). In the capital Manaus, the months from January to July signaled mild stress, but from August to November, there was moderate stress, and in December, there was mild stress (*p* < 0.05).

**Figure 4:**
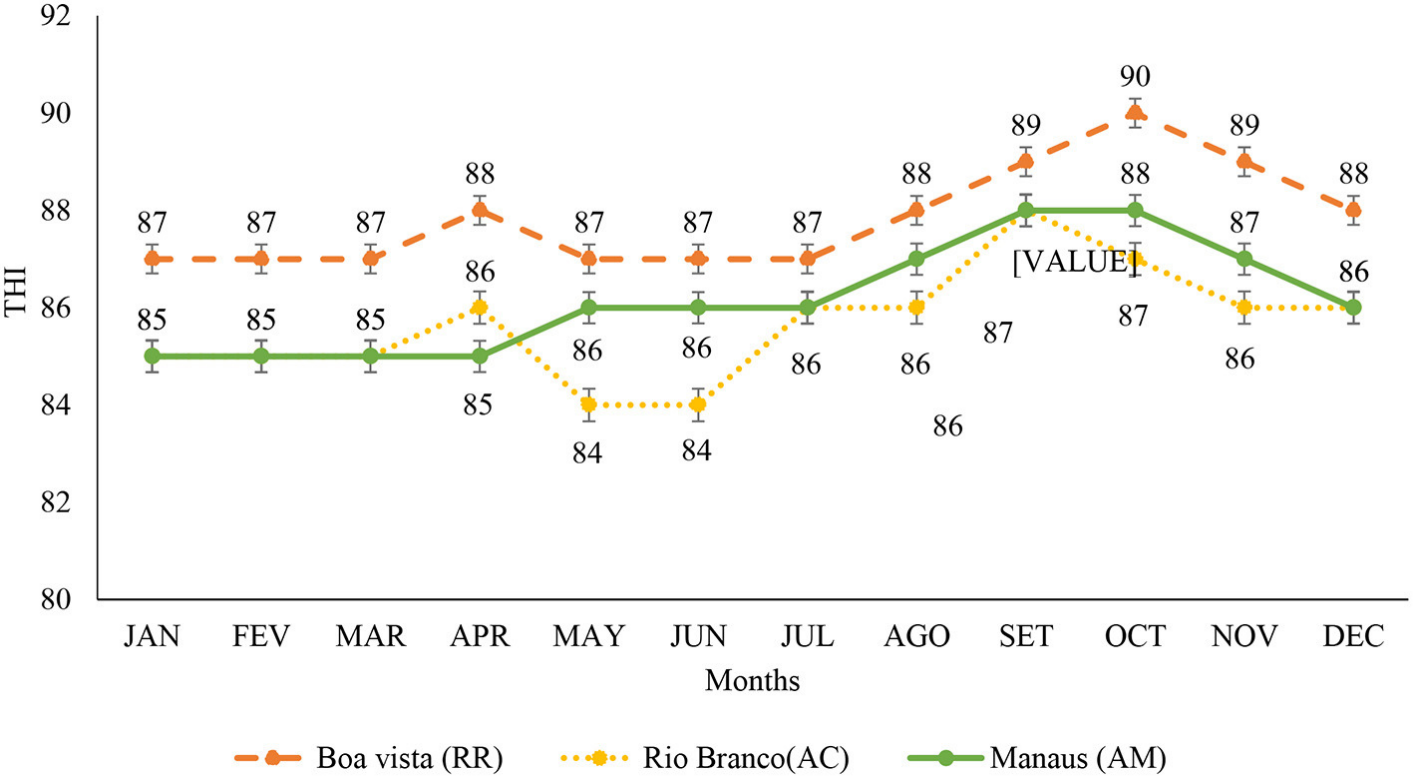
Maximum temperature and humidity index from January to December in the capitals of Roraima, Rio Branco, and Manaus, for the period from 1993 to 2020. <72 indicates without stress; 72–78 indicates mild or mild stress; 79–88 indicates moderate stress; 89–98 indicates severe stress (26). JAN, January; FEB, February; MAR, March; APR, April; MAY, May; JUN, June; JUL, July; AGO, August; SET, September; OCT, October; NOV, November; and DEC, December.

In Figure 4, the maximum THI is observed, indicating that the three capitals under study were under thermal stress in all months of the year, and classified as moderate and severe stress. In Boa Vista, the months from January to August were indicative of moderate stress, from September to November indicated severe stress, and December indicated moderate stress. The capitals Rio Branco and Manaus presented moderate stress without classification variation in all months of the year, with the months of September, October, November, and December showing the highest rates of THI.

Regarding the minimum THI for the years 1993 to 2020, the data presented in Table 1 shows that according to the classification described by Armstrong (26), the capital of Boa Vista presents a THI variation that indicates animals without thermal stress and with mild stress among those mentioned similar to data from the capital Manaus. In Boa Vista, there was mild thermal stress in all years of study. In the capital of Rio Branco, the THI indicated thermal comfort in all years, animals without stress, with the lowest THI being in 1993 and the highest THI in 2015. It was observed that all the capitals under study had an increase in THI between the years 2011 to 2020. In the capital Manaus, the years 1993, 1998, 1999, and 2000 presented thermal comfort, with the other years of study classified as mild thermal stress.

**Table 1:** Minimum THI of the capitals Boa Vista, Rio Branco, and Manaus from 1993 to 2020.

**Year**	**Boa Vista (RR)**	**Rio Branco (AC)**	**Manaus (AM)**
1993	73	67	71
1994	73	68	72
1995	73	68	72
1996	73	68	72
1997	74	70	72
1998	73	69	71
1999	72	68	69
2000	72	68	70
2001	72	68	72
2002	72	69	73
2003	71	68	73
2004	72	69	73
2005	73	68	73
2006	73	69	73
2007	73	69	73
2008	73	68	72
2009	73	69	73
2010	73	68	74
2011	73	69	73
2012	73	69	74
2013	73	69	74
2014	73	70	74
2015	74	71	75
2016	74	70	75
2017	74	70	74
2018	73	69	74
2019	73	70	74
2020	73	70	74

< 72 indicates without stress (green color); 72–78 indicates mild or mild stress (yellow color) (26).

In the analysis of the mean annual THI, heat stress indices were shown in all three capitals in all years of study (Table 2). In Boa Vista, according to Armstrong's classification (26), it was noted that the years 1999 to 2004, 2007, 2008, and 2012 presented mild thermal stress and the other years had THI above 78, indicating moderate thermal stress. The capital of Rio Branco maintained the average THI below 78 throughout the studied series, presenting mild thermal stress, unlike the capital of Manaus, which showed mild thermal stress indices from 1993 to 2002, with an increase in the average THI being observed in the years from 2003 to 2005, classified as moderate thermal stress; however, the years 2006, 2007, 2008, 2011, and 2012 showed a reduction in the THI, returning to the classification of mild thermal stress. On the other hand, the years 2013 to 2020 had an increase in THI, classified as moderate thermal stress. In this perspective, the capital of Rio Branco maintained an average THI lower than (79) throughout the years, always remaining in mild thermal stress, unlike the capitals, Boa Vista and Manaus, which presented THI variations in mild and moderate stress, respectively, with Boa Vista showing the highest average THI specifically in the year 1997 (81).

**Table 2:** Average THI of the capitals Boa Vista, Rio Branco, and Manaus from 1993 to 2020.

**Year**	**Boa Vista (RR)**	**Rio Branco (AC)**	**Manaus (AM)**
1993	79	75	77
1994	79	76	77
1995	80	75	78
1996	80	76	77
1997	81	76	78
1998	80	76	78
1999	78	75	77
2000	78	75	77
2001	78	76	78
2002	78	76	78
2003	78	75	79
2004	78	75	79
2005	79	75	79
2006	79	75	78
2007	78	75	78
2008	78	75	78
2009	79	76	79
2010	79	75	79
2011	79	75	78
2012	78	76	78
2013	79	76	79
2014	79	76	79
2015	80	77	79
2016	80	76	79
2017	80	76	79
2018	79	76	79
2019	79	76	79
2020	79	76	79

72–78 indicates mild or mild stress (yellow color); 79–88 indicates moderate stress (orange color) (26).

Regarding the maximum THI, thermal stress was observed in all the capitals of this study during all the years of the historical series, the capitals Rio Branco and Manaus did not show any differences (*p* > 0.05) in the THI, remaining in all the years in moderate thermal stress, being (84) the lowest THI obtained in the capitals in the years 1995 and 2008 in Rio Branco, and in the year 1993 in Manaus, in both capitals the maximum THI obtained remained between (86) and (87), unlike Boa Vista, which showed a difference (*p* < 0.05) in the maximum THI compared to the other capitals in the study, with severe heat stress indices from 2015 to 2018 (Table 3).

**Table 3:** Maximum THI of the capitals Boa Vista, Rio Branco, and Manaus from 1993 to 2020.

**Year**	**Boa Vista (RR)**	**Rio Branco (AC)**	**Manaus (AM)**
1993	87	85	84
1994	87	83	85
1995	88	84	85
1996	88	83	85
1997	88	86	86
1998	88	86	87
1999	88	85	86
2000	86	85	87
2001	86	86	86
2002	86	86	86
2003	87	86	87
2004	87	86	87
2005	87	86	88
2006	87	86	87
2007	87	86	87
2008	88	84	87
2009	88	87	87
2010	88	87	87
2011	88	86	86
2012	88	86	86
2013	88	86	86
2014	88	86	86
2015	89	87	87
2016	89	87	86
2017	89	87	86
2018	89	86	86
2019	88	87	86
2020	88	87	86

79–88 indicates moderate stress (orange color); 89–98 indicates severe stress (red color) (26).

**Figure 5:**
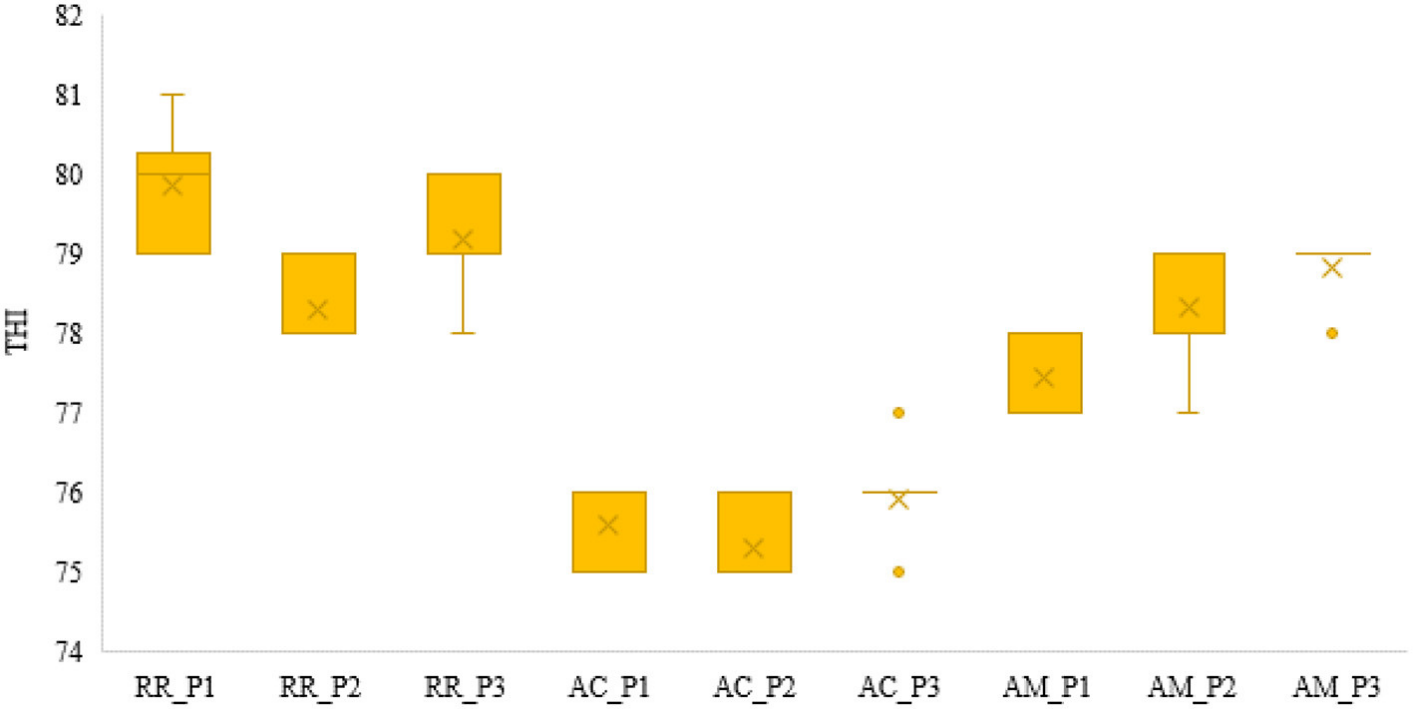
Box plot of average THI by states (RR, Roraima; AC, Acre; and AM, Amazonas) in each period (P1 = 1993 to 1999; P2 = 2000 to 2009, and P3 = 2010 to 2020).

Figure 5 shows the minimum, average, and maximum THI (standard deviation) with comparisons between states (Roraima, Acre, and Amazonas), and it was possible to observe differences between states by period and between states (*p* < 0.05). In addition, it is possible to notice higher THI indices in Roraima (RR), followed by Manaus (AM), and Rio Branco (AC) having the lowest index.

## 4. Discussion

The results obtained from the estimation of data for the evaluation of thermal stress in ruminants using the values of equation 1 adapted from (25) referring to the minimum, medium, and maximum THI for all months of the year in the capitals of Roraima, Acre, and Amazonas demonstrated that there were variations in the classification of the THI according to the model proposed by Armstrong (26).

In the data presented for minimum THI (Table 1), it was found that in Rio Branco, there was thermal comfort in all months of the year; on the other hand, in the capitals Boa Vista and Manaus, there was a sign of reduced thermal comfort, being presented in most of the months of the year as a mild stress rating. Costa et al. (28) and Polli et al. (8) highlighted that even with a minimum THI and regardless of the season of the year, the wind speed plays a fundamental role in thermal comfort, promoting the Santa Inês sheep heat loss through convection (29); therefore, when changes occur, these climatic factors act directly or indirectly on the animals, taking them out of their comfort zone, causing a state of stress, even with a favorable temperature and season.

In this context, heat stress occurs when animals are exposed to extreme temperatures, which are beyond their comfort zone. This can lead to a range of physiological and behavioral responses that affect the welfare and health of the animals (15). Environmental conditions play an important role in the response to thermal stress. Ambient air, for example, can influence the ability of animals to dissipate body heat. Elevated temperatures and high levels of humidity make it difficult to lose heat through perspiration and heat exchange with the environment. Direct sunlight can also increase the thermal load on animals, especially when shaded areas are not available (19, 30–32).

On the other hand, animals placed in environments classified as having a high THI have difficulty dissipating heat as they are subjected to a temperature above tolerable, suffering thermal stress as a consequence, and the endogenous heat production is greater than the cooling capacity; thus, the heat shock causes an increase in body temperature, causing indices above reference values (33–38). Thus, it was noted in this study that the average THI (Table 2) in Boa Vista and Manaus showed negative asymmetry with 72–78 indicating mild stress and 79–88 indicating moderate stress, and in Rio Branco, there was evidence of moderate stress in all months of the year. Azevêdo et al. (39) argued that animals intended for reproduction when exposed to this classification of THI may have impairment in their reproductive capacity, especially in a situation of thermal stress resulting from the effects of THI above 72, according to the result found in this study.

In a study carried out by Costa et al. (28) in the municipality of Porto Velho-RO, which has a dry and humid tropical climate with characteristics similar to those of the regions studied, there was an indication of severe thermal stress for animals exposed to the sun at certain times of the day, and this can be related to high humidity and low wind speed in the region, affecting herd productivity indices, corroborating the results of this research, where the maximum THI (Table 3) signaled moderate stress for the capitals Rio Branco and Manaus (79–88) and for Boa Vista indicated moderate stress (79–88) and severe stress (89–98).

When analyzing the minimum, average, and maximum THI data, it is possible to analyze variations in their values, and these results may indicate climatic trends that have occurred in recent years, similar to those found by Dantas et al. (40), who sought to evaluate trends in climate data between the years 1991 and 2004, where he identified climatological changes capable of altering the THI and, consequently, causing thermal stress in production animals.

In the capital Boa Vista, the months of January, February, May, June, and July showed an indication of mild stress, and the months of March, April, August, September, October, November, and December had moderate stress. In Rio Branco, all months of the year presented the average THI in mild stress with variations of lower THI (73) and higher THI (77). In the capital Manaus, the months from January to July signaled mild stress, but from August to November, there was moderate stress, and in December, there was mild stress. In this way, the study of climatic variables in the Amazon region presents stressful climatological variations; since they have high temperatures and high relative humidity during the course of the year, this difference was notorious when compared to other Brazilian regions (41). Therefore, in this study, it is possible to show significant variations in THI elevation, without a decrease in the last 10 years from 2010 to 2020 (40). In the municipality of Humaitá (AM), in the month of January, the rainiest period in the region, a THI of (75) was observed, demonstrating a range of mild stress, but considered an alert situation for dairy cattle.

Rosanova et al. (42) identified THI between 75 and 78 in the northern region of the State of Tocantins, which can collaborate with the implementation of strategies that can alleviate the thermal stress of the bovine, in the context of handling, wellbeing, and animal behavior, and these results were similar to the findings of this study. In another research carried out by Lima et al. (43), in Barbalha, Ceará, the THI averages concentrated between 77 and 82, in the months of November, were considered a situation of mild stress (72–82) and moderate stress (79–88). In the months from January to April, the THI was concentrated in the range of (76) and classified as a mild or mild stress environment, corroborating the results found in the minimum THI classification for the city of Manaus.

The maximum THI results present in the three municipalities of the Western Amazon are similar to those found by Lima et al. (44), who identified a THI of above 80 from August to September for the city of Amapá, which is a value denoting moderate stress, noting that these high THI conditions tend to influence the reduction of animal fattening and increased frequency respiratory and stress in cattle.

In an experiment carried out in the municipality of Sena Madureira-Acre, from January to March 2020, it was observed that the THI values were ≥72, and a similar result was also evidenced in this study in Rio Branco (Acre), which indicated a minimum THI of 72 in January (45). According to Soares et al. (46), the high rates of THI favor the stimulus of thermal stress and, consequently, influence the reduction of milk production, reproduction, and low animal welfare performance in dairy cows (15, 31).

Information similar to that alerted in this study, in a research study in the municipality of Humaitá-AM, in the Western Amazon, Rohleder et al. (47) identified a state of thermal stress during dairy holidays, classified as alert, with THI values ranging from 56.25 (comfortable) to 84.68 (emergency) in August and from 74.15 (comfortable) to 84.07 (emergency) in January.

Different strategies must be adopted according to the purpose of producing the species used in the region. Thus, we suggest different strategies for ruminants destined for beef and milk production since in most of the years and months presented, the heat stress indices are evident and can strongly corroborate the reduction in the productive parameters of the animals.

Based on the information described above, in the capitals studied, to minimize heat stress in ruminants raised in the field during the hottest hours of the day, we suggest adopting the following strategies:

### 4.1. Provide shade

Make sure there are shaded areas available on the pasture where the cattle can take shelter from the direct sun. This can be done through trees, artificial shelters, or shade structures.

### 4.2. Provide fresh water

Keep drinkers or water tanks clean and have fresh water available at all times. Cattle need enough water to hydrate and regulate their body temperature.

### 4.3. Proper feed

Offer a balanced, high-quality diet for cattle. The feed should be rich in nutrients, providing enough energy to cope with heat stress. Consider providing additional dietary supplements, such as mineral salts, which help regulate body temperature.

### 4.4. Pasture management

Adopt appropriate management practices for the pasture, such as pasture rotation. This allows cattle access to areas with fresh pasture and prevents overgrazing, which can aggravate heat stress.

### 4.5. Feeding times

Adjust feeding times for cooler times of the day, such as early morning or evening when temperatures are cooler. This helps to avoid overexposure to heat during digestion.

### 4.6. Spraying or water baths

Install water spray systems or allow cattle to take water baths to cool off. This can help reduce body temperature and heat stress.

### 4.7. Health monitoring

Be aware of signs of heat stress such as rapid breathing, excessive salivation, reduced food intake, and agitated behavior.

## 5. Conclusion

The analysis of the thermal comfort index in the capitals Boa Vista, Rio Branco, and Manaus over the 27 years of the study showed notable variations in maximum, medium, and minimum THI, with THI of 72, signaling moderate or severe thermal stress, with a gradual increase in temperature and humidity indices in the last 10 years. In this perspective, the creation of ruminants in the mentioned capitals is challenging due to the influence of thermal stress, which can affect the characteristics of the production and reproduction of these animals. Therefore, the adoption of trees, as well as the implementation of artificial shading, can be a fundamental strategy in order to reduce thermal stress indices in animals raised in this region. In addition to this, the supply of water to these animals becomes essential due to the strong heat waves and thermal stress in the region.

## Data availability statement

The raw data supporting the conclusions of this article will be made available by the authors, without undue reservation.

## Author contributions

WS and JL-J: experiment design and original writing. WS, OP, DL, ÉS, MS, RC, AB, JS, AS, LS, and JL-J: experiment execution and investigation. WS and AB: data curation and formal analysis. CA and EB: conceptualization, data curation, and writing—original draft preparation and investigation. All authors edited and approved the final manuscript.
